# Detection and Characterization of Extended‐Spectrum Beta‐Lactamase‐Producing *Escherichia coli* in Raw Seafood From the Coastal Area of Bangladesh

**DOI:** 10.1002/mbo3.70023

**Published:** 2025-06-27

**Authors:** Zannatul Firdous, Md. Saiful Islam, Md. Ashek Ullah, Md. Liton Rana, Farhana Binte Ferdous, Al‐Muksit Mohammad Taufiquer Rahman, Jayedul Hassan, Md. Tanvir Rahman

**Affiliations:** ^1^ Department of Microbiology and Hygiene, Faculty of Veterinary Science Bangladesh Agricultural University Mymensingh Bangladesh; ^2^ Department of Animal Science University of California—Davis Davis California USA; ^3^ University of Chinese Academy of Sciences Beijing China; ^4^ Department of Medicine Rajshahi Medical College Rajshahi Bangladesh

**Keywords:** antimicrobial resistance, Bangladesh, beta‐lactams, *E. coli*, ESBL, public health, seafood

## Abstract

The emergence of antimicrobial resistance (AMR) and extended‐spectrum beta‐lactamase (ESBL)‐producing *Escherichia coli* (ESBL‐EC) in seafood represents a significant public health concern. In this study, we screened 102 raw seafood samples, comprising shrimp (*n* = 42), sea fish (*n* = 36), and crabs (*n* = 24), to detect ESBL‐EC. *E. coli* was isolated and identified through culture‐based methods, staining procedures, biochemical assays, and polymerase chain reaction (PCR) analysis. The AMR properties of *E. coli* isolates were evaluated using the disc diffusion test, while ESBL‐EC was identified phenotypically through the double‐disc synergy test and confirmed at the genetic level using PCR. PCR analysis revealed that 42.2% (43/102) of the samples were contaminated with *E. coli*, with sea fish showing the highest (*p* < 0.05) prevalence (63.9%, 23/36), followed by crabs (37.5%, 9/24) and shrimp (26.2%, 11/42). All the isolates exhibited phenotypic resistance to ampicillin, followed by ceftazidime (95.3%), ciprofloxacin (55.8%), azithromycin (39.5%), cefotaxime (37.2%), and streptomycin (16.3%). Notably, 69.8% (30/43) of *E. coli* isolates exhibited phenotypically multidrug resistance. Moreover, 18.6% (8/43) of the isolates showed ESBL‐producing characteristics, higher in shrimp than in sea fish and crabs. ESBL‐related gene, *bla*_TEM_, was detected in 75% (6/8), *bla*_SHV_ in 87.5% (7/8), and *bla*_CTX‐M_ in 50% (4/8) of the ESBL‐EC isolates. Regular surveillance of seafood for antimicrobial‐resistant bacteria, particularly ESBL‐producing strains, is recommended due to their potential public health implications.

## Graphical abstract

This study determined the presence of extended‐spectrum beta‐lactamase‐producing *Escherichia coli* (ESBL‐EC) in seafood samples in Bangladesh. The findings also indicated that ESBL‐EC showed higher phenotypic resistance profiles and contained ESBL‐related resistance genes.

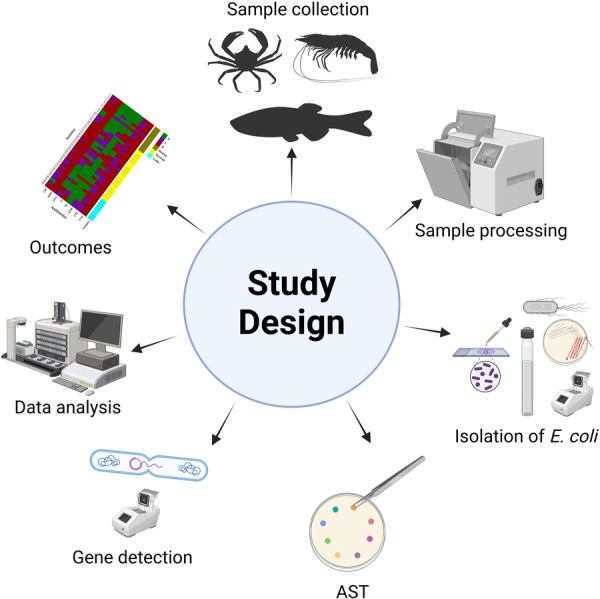

## 1 Introduction

For many coastal communities, seafood has been a fundamental component of the diet, offering vital nutrients, including easily digestible proteins (Huss 1993; Faber et al. 2010). Seafood is projected to surpass poultry in driving the expansion of the global protein supply, becoming the leading contributor to overall protein growth worldwide (Robobank 2025). Worldwide demand for seafood is anticipated to increase at an average annual rate of 5%, reflecting a steady upward trend in consumption patterns across global markets (Global Seafoods 2025). Bangladesh ranked among the top fish‐producing nations in 2022–2023, reaching a total output of 4.915 million metric tons. This significant achievement enabled the country to attain self‐sufficiency in fish production, with an average daily per capita fish intake of 67.8 g (Department of Fisheries 2023). Seafood plays a crucial role in Bangladesh's nutritional security, job creation, and international trade. Seafood ranks 3rd in both inland fisheries capture and aquaculture production, contributing to 3.57% of the country's GDP and 1.24% of total export earnings (Department of Fisheries 2024). However, seafood can also act as a vector for infections and foodborne illnesses, underscoring the need for stringent bacteriological monitoring (Costa 2013).

*Escherichia coli*, naturally present in the intestinal tracts of both humans and animals, is increasingly recognized as a concern in seafood safety. This bacterium is frequently implicated in seafood contamination, often causing infections when contaminated seafood products are consumed, particularly in tropical regions (Costa 2013). Although several strains of *E. coli* are non‐pathogenic and play beneficial roles, some pathogenic variants can induce serious gastrointestinal issues, kidney complications, or even critical health conditions in both humans and animals (Costa 2013; Beauchamp and Sofos 2010; Surendraraj et al. 2010). Contamination in seafood often arises from exposure to water polluted by human or animal waste, inadequate sanitation during processing, or cross‐contamination during storage and handling (Brauge et al. 2024). Additionally, improper cooking or handling practices can exacerbate the risks of *E. coli* transmission to consumers (Loest et al. 2022).

The rise of antimicrobial resistance (AMR) has become a significant challenge to global health security in the 21st century, impacting all regions worldwide (WHO 2014). Bangladesh, a growing Southeast Asian country with a high incidence of AMR, triggers both global and regional threats (Ahmed et al. 2019). Furthermore, the rising resistance of *E. coli* to various antibiotic groups makes these infections troublesome to treat. Antibiotic‐resistant bacteria have spread to numerous environments due to the frequent application of antimicrobial agents in hospitals, livestock, agriculture, and fishing industries (Kümmerer 2009; Yang et al. 2013). In recent decades, the occurrence of AMR in *E. coli* has risen dramatically. A majority of *E. coli* strains produce beta‐lactamase enzymes, which degrade the beta‐lactam ring, leading to antibiotic ineffectiveness. The presence of extended‐spectrum beta‐lactamase‐producing *E. coli* (ESBL‐EC) serves as an indicator of the environmental development of multidrug‐resistant (MDR) bacterial isolates. The CTX‐M, TEM, and SHV beta‐lactamases are considered clinically significant worldwide (Islam et al. 2022). However, ESBL‐EC poses a major risk to both human and animal health, reducing the effectiveness of available antibiotic treatments (Department of Fisheries 2024).

The presence of MDR bacteria in fish can endanger public health because fish are thought to be potential carriers of foodborne bacterial diseases (Hassen et al. 2020). Despite its relevance to public health, we are still unclear about any molecular evidence‐based data on ESBL‐EC from seafood in Bangladesh. Moreover, it is essential to be up to date with the most recent data available to mitigate the effects of ESBL‐EC on health systems, the environment, and food chains. Therefore, the objectives of this study were: (1) to isolate *E. coli* from raw seafood samples collected from coastal markets in Bangladesh, (2) to characterize the antimicrobial resistance profiles of these isolates, and (3) to identify and confirm the presence of ESBL‐EC using phenotypic and genotypic techniques.

## 2 Materials and Methods

### 2.1 Ethical Statement

All study procedures were approved by the Animal Welfare and Ethics Committee of Bangladesh Agricultural University (BAU), Mymensingh, Bangladesh (Approval Number: AWEEC/BAU/2023(25)). Verbal consent was obtained from vendors before collecting samples.

### 2.2 Sample Collection and Processing

**Figure 1:**
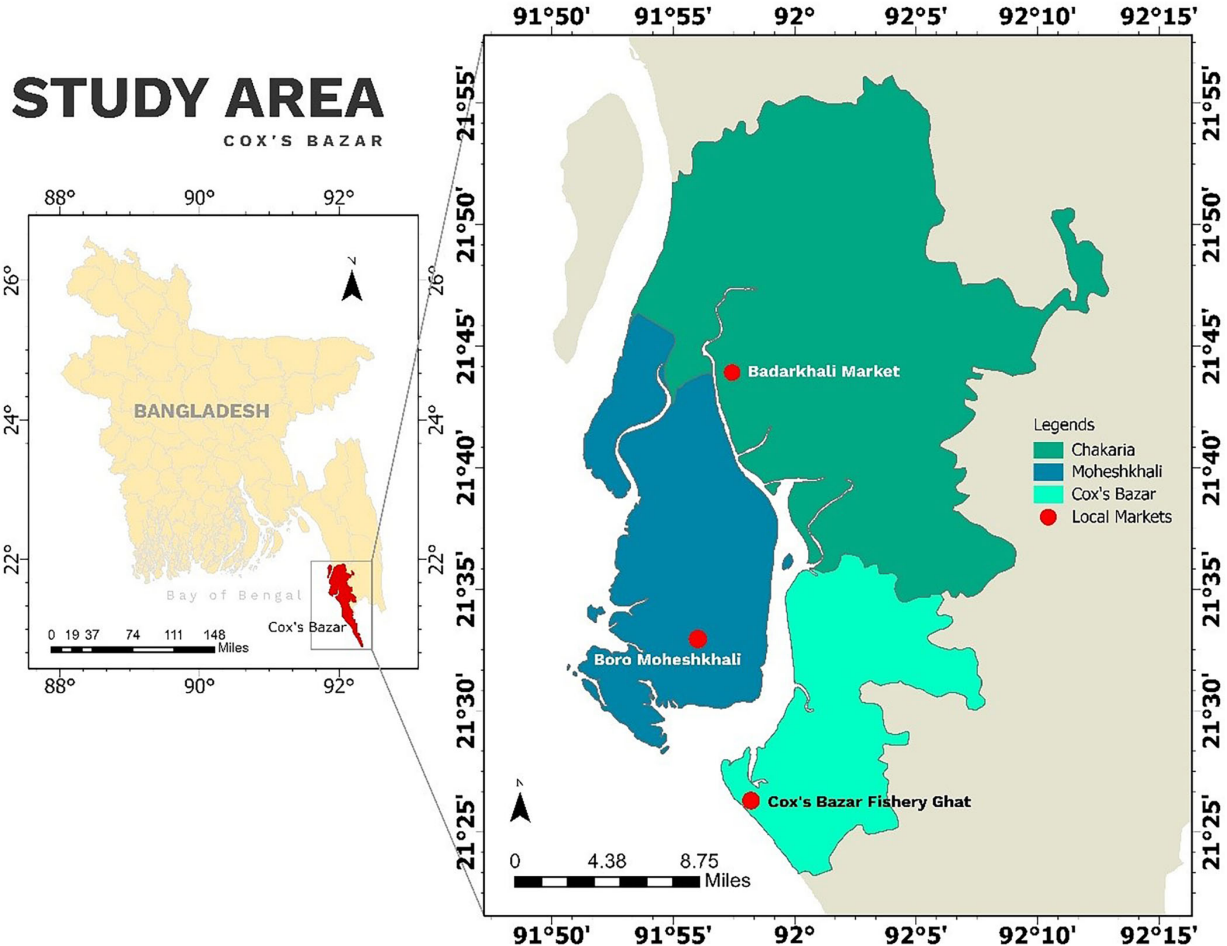
Map of the study area, developed using ArcMap 10.7 (ESRI, USA) with the aid of Geographic Information System (GIS).

This study targeted the coastal areas of the Chattogram Division in Bangladesh, including Chakaria, Moheshkhali, and Cox's Bazar Sadar upazilas (Figure 1). The study areas were selected considering the availability and popularity of seafood in that region. In total, 102 raw seafood samples were obtained from various open markets across the selected sites between July and December 2022. Five different types of seafood samples, e.g., white shrimp/common prawn (*Palaemon serratus*, *n* = 26), tiger prawn (*Penaeus monodon*, *n* = 16), tuna fish (*Thunnus* spp., *n* = 12), pomfret fish (*Pampus argenteus*, *n* = 24), and crab (*Portunus sanguinolentus*, *n* = 24) were used for the study, where white shrimp and tiger prawn are grouped as “Shrimp,” tuna fish, and pomfret fish are grouped as “Sea fish.” The number and type of seafood samples were determined based on availability, consumer popularity, and logistical feasibility across multiple markets during the study period. Efforts were made to capture a representative snapshot of commonly consumed seafood in the selected coastal regions.

Sample collection was carried out at multiple retail fish markets every 2 weeks over the duration of the study. The study exclusively included fresh and unprocessed samples, ensuring that none were frozen or pretreated. Any fish showing apparent signs of disease or physical damage were omitted from the study. Medium‐sized individuals of each species were selected to reduce variability and improve comparability. This size criterion balanced biological maturity and sample consistency while reflecting typical market preferences. All samples were carefully selected and collected by a team consisting of two experienced veterinarians and two skilled microbiologists. All collected samples were promptly sealed in sterile zipper bags and kept on ice for transport to the laboratory.

At the laboratory, samples were handled under aseptic conditions to prevent cross‐contamination during processing. The external surfaces of the seafood were gently wiped with 70% (v/v) alcohol, not for disinfection purposes, reflective of consumer handling, but to minimize external contamination and ensure that the microbiological analyses focused on internal tissues rather than surface‐associated microbes introduced during transportation or handling. A 25 g portion of seafish samples was blended with 225 mL of buffered peptone water (BPW) broth (HiMedia, Mumbai, Maharashtra, India) and incubated overnight at 37°C (Ullah et al. 2024). In the case of shrimp and crabs, the brain, legs, muscles, and intestines were combined and homogenized, with a 25 g portion being prepared, added to BPW broth, and incubated under the same conditions (Ullah et al. 2023; Haque et al. 2023).

### 2.3 Isolation and Molecular Detection of *Escherichia coli*

Following overnight enrichment, a small amount of the sample (10 µL) was taken using a loop and inoculated onto an eosin methylene blue agar plate (HiMedia, Mumbai, Maharashtra, India). The inoculated plate was incubated at 37°C for 24 h to facilitate bacterial growth. Suspected colonies were underwent to Gram staining and biochemical assays for additional confirmation (Hitchins et al. 1998), followed by molecular detection by polymerase chain reaction (PCR) (Table 1). The PCR‐positive controls encompassed *E. coli* genomic DNA that had previously tested positive for the relevant genes. For PCR‐negative controls, non‐template controls were implemented, employing sterile phosphate‐buffered solution instead of genomic DNA. We took one isolate per sample.

**Table 1:** List of primers utilized in this study, along with their respective sequences and amplicon sizes.

Target genes	Primer sequence (5′–3′)	Amplicon size (bp)	Annealing temperature	References
mal*B*	F: GACCTCGGTTTAGTTCACAGA	585	58	Wang et al. (1996)
	R: CACACGCTGACGCTGACCA			
*bla*_TEM_	F: CAGCGGTAAGATCCTTGAGA	643	55	Chen et al. (2004)
	R: ACTCCCCGTCGTGTAGATAA			
*bla*_SHV_	F: GGCCGCGTAGGCATGATAGA	714	55	
	R: CCCGGCGATTTGCTGATTTC			
*bla*_CTX‐M_	F: ACGCTGTTGTTAGGAAGTG	857	58	Omati et al. (2016)
	R: TTGAGGCTGGGTGAAGT			

Genomic DNA for PCR was prepared as a crude lysate using a heat lysis method (Ferdous et al. 2023). Briefly, 1 mL of an overnight bacterial culture was centrifuged at 2300 × g for 5 min using a KUBOTA 6500 centrifuge (Japan). The supernatant was discarded, and the bacterial pellet was resuspended in 200 μL of phosphate‐buffered saline. The suspension was heated at 98°C for 10 min in a water bath to lyse the cells, then allowed to cool in a cold chain. After cooling, the lysate was centrifuged at 9200 × g for 10 min, and the resulting supernatant, containing crude genomic DNA, was transferred to sterile Eppendorf tubes (HiMedia, Mumbai, Maharashtra, India) and stored at −20°C for PCR use.

### 2.4 Antibiotic Susceptibility Test

To evaluate the AMR patterns of the *E. coli* isolates, the Kirby‐Bauer disc diffusion technique (DDT) (Bauer et al. 1966) was employed, following the recommendations provided by the Clinical and Laboratory Standards Institute (CLSI) (CLSI 2022). Eight antibiotics (HiMedia, Mumbai, Maharashtra, India) representing six antimicrobial categories commonly utilized in human health, veterinary practice, and aquaculture in Bangladesh were tested. The antibiotics used were ciprofloxacin (5 µg), gentamicin (10 µg), tetracycline (30 µg), streptomycin (10 µg), ampicillin (10 µg), cefotaxime (30 µg), and ceftazidime (30 µg). Antimicrobial susceptibility for azithromycin was checked using the broth dilution method following the National Antimicrobial Resistance Monitoring System (NARMS) guidelines (CDC 2025). After incubation for 16–18 h on EMB agar, 2–3 colonies were suspended in 0.85% sterile saline solution and adjusted to match the 0.5 McFarland turbidity standard. The standardized bacterial suspension was then uniformly spread onto Mueller‐Hinton agar (HiMedia, Mumbai, Maharashtra, India) using sterile cotton swabs. This was followed by incubation at 37°C for 24 h. Any isolates demonstrating resistance to at least three distinct antibiotic classes were categorized as multidrug‐resistant (MDR) (Magiorakos et al. 2012). The multiple antibiotic resistance (MAR) index was computed using the formula as previously described (Krumperman 1983).

### 2.5 Phenotypic and Genotypic Detection of ESBL‐Producing *Escherichia coli*

Phenotypic identification of ESBL‐EC was performed using the double‐disc synergy test, following the method described by Islam et al. (Wang et al. 1996). ESBL‐EC isolates confirmed through phenotypic testing were further examined for the presence of beta‐lactamase genes. The genes *bla*_TEM_, *bla*_CTX‐M_, and *bla*_SHV_ were detected using simplex PCR (Table 1).

### 2.6 Statistical Analysis

The collected data for this study were managed using Excel 365 (Microsoft/Office 365, Redmond, WA, USA) and analyzed with the Statistical Package for Social Sciences (SPSS, version 25, IBM, Chicago, IL, USA) and GraphPad Prism (version 8.4.2, San Diego, CA, USA). Descriptive statistical methods were used to examine the distribution of different variables. The prevalence was estimated by calculating a binomial 95% confidence interval (CI_95%_), following an established approach (Brown et al. 2001), which was executed using GraphPad Prism. Additionally, a chi‐square test (proportional Z‐test) was performed to assess the variation in the frequencies of *E. coli* isolates. A bivariate analysis incorporating the Spearman correlation test was conducted to evaluate the relationship between the resistance profiles of *E. coli* isolates. A *p*‐value of less than 0.05 was considered statistically significant.

## 3 Results

### 3.1 Occurrence of *Escherichia coli*

Out of 102 raw seafood samples, *E. coli* was detected in 42.2% (CI_95%_: 33.0; 51.9) of samples using the molecular method. The occurrence of *E. coli* differs significantly among the samples. Shrimp, sea fish, and crab had detection rates of 26.2% (11/42, CI_95%_: 15.3; 41.1), 63.9% (23/36, CI_95%_: 47.6; 77.5), and 37.5% (9/24, CI_95%_: 21.2–57.3), respectively. In shrimp types, 30.7% (8/26, CI_95%_: 16.5; 49.9) of white shrimp and 18.7% (3/16, CI_95%_: 6.6; 43.0) of tiger prawn harbored *E. coli*. Moreover, *E. coli* was found in 91.6% (11/12, CI_95%_: 64.6; 99.6) of tuna fish and 50% (12/24, CI_95%_: 31.4; 68.6) of pomfret fish.

### 3.2 Antibiogram of Isolated *Escherichia coli*

**Figure 2:**
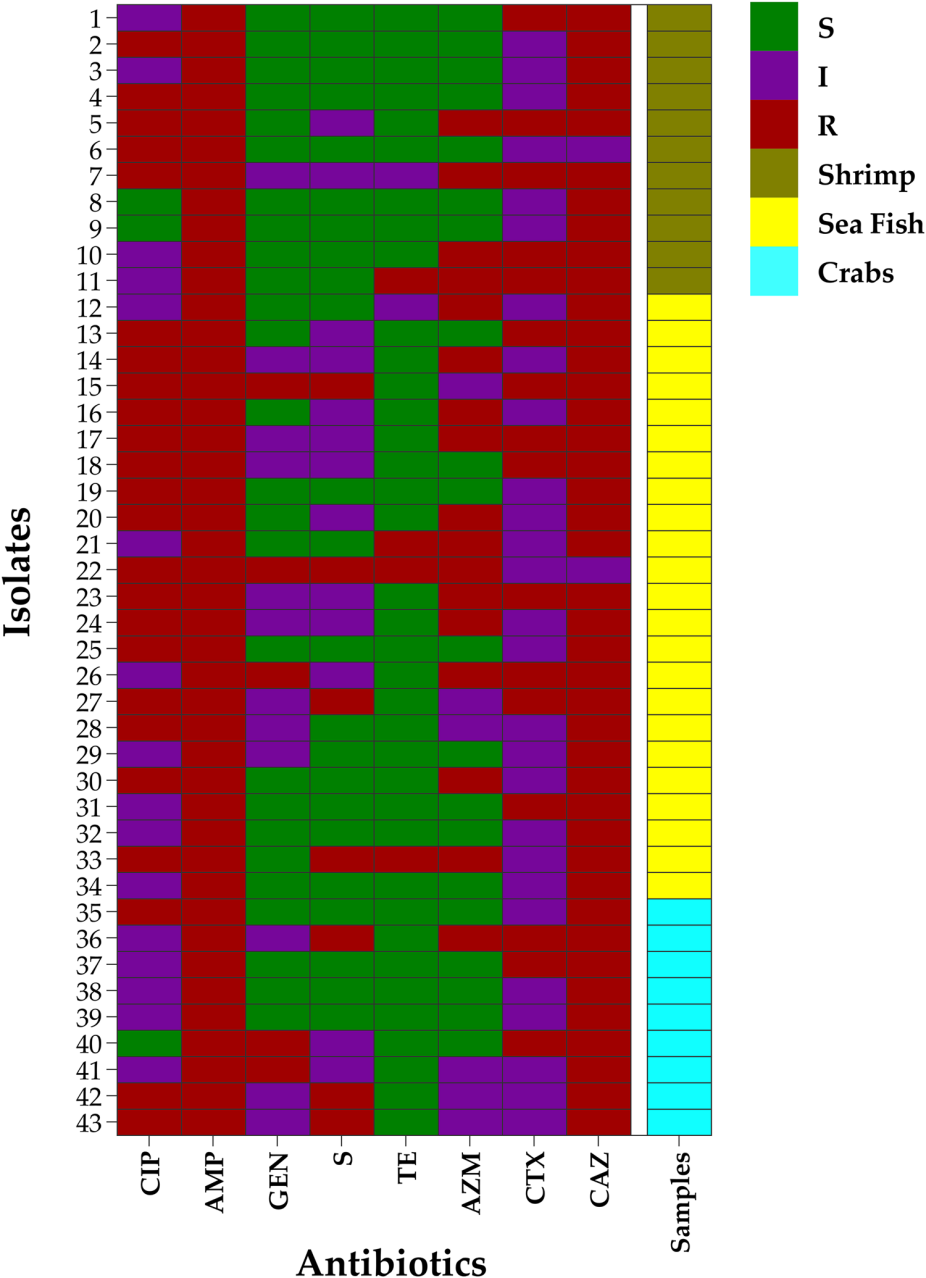
The resistance patterns of the individual *E. coli* isolates from raw seafood. AMP, ampicillin; CAZ, ceftazidime; CIP, ciprofloxacin; CTX, cefotaxime; GEN, gentamicin; S, streptomycin; TE, tetracycline.

In DDT, all the 43 *E. coli* isolates were resistant to ampicillin (100%), followed by ceftazidime (95.4%, 41/43, CI_95%_: 84.5; 99.2), ciprofloxacin (55.8%, 24/43, CI_95%_: 41.1; 69.6), azithromycin (39.5%, 17/43, CI_95%_: 26.4; 54.4), cefotaxime (37.2%, 16/43, CI_95%_: 24.4; 52.1), streptomycin (16.3%, 7/43, CI_95%_: 8.1; 29.9), gentamicin (11.6%, 5/43, CI_95%_: 5.1; 24.5), and tetracycline (9.3%, 4/43, CI_95%_: 3.7; 21.6) (Figure 2).

By bivariate analysis, a significant, strong, and positive correlation was noted between azithromycin and tetracycline resistance patterns (Spearman correlation coefficients, *ρ* = 0.396, *p* = 0.009) (Table 2).

**Table 2:** Spearman correlation coefficients assessing associations between antibiotic resistance in *E. coli* isolates from seafood samples.

		CIP	AMP	GEN	S	TE	CTX	AZM	CAZ
CIP	SC value	1							
	*p*‐value								
AMP	SC value	—	—						
	*p*‐value	—	—						
GEN	SC value	−0.116	—	1					
	*p*‐value	0.461	—						
S	SC value	0.265	—	0.233	1				
	*p*‐value	0.085	—	0.133					
TE	SC value	−0.037	—	0.134	0.293	1			
	*p*‐value	0.811	—	0.393	0.057				
CTX	SC value	−0.09	—	0.171	0.052	−0.081	1		
	*p*‐value	0.565	—	0.273	0.743	0.606			
AZM	SC value	0.145	—	0.003	0.03	0.396**	0.165	1	
	*p*‐value	0.354	—	0.982	0.849	0.009	0.291		
CAZ	SC value	−0.244	—	−0.185	−0.127	−0.227	0.022	0.035	1
	*p*‐value	0.115	—	0.234	0.419	0.144	0.889	0.825	

*Note:* Here, the significance codes: “**” at 0.01. Where the value was not computed (because at least one of the variables is constant).

Abbreviations: AMP, ampicillin; CAZ, ceftazidime; CIP, ciprofloxacin, CTX, cefotaxime; GEN, gentamicin; SC, spearman correlation coefficient; S, streptomycin; TE, tetracycline.

Thirty *E. coli* isolates (69.8%, CI_95%_: 54.9; 81.4) were MDR, where the MAR indices varied from 0.25 to 0.75. The most common MDR patterns were CIP‐AMP‐CAZ (*n* = 6) and CIP‐AMP‐AZM‐CAZ (*n* = 5). Two isolates exhibited resistance to six antibiotics from five classes (Table 3).

**Table 3:** Multidrug resistance and multiple antibiotic resistance profiles of *Escherichia coli*.

No.	Resistance pattern	No. of antibiotics (classes)	No. of isolates	Overall MDR (%)	MAR index
1	CIP, AMP, GEN, S, CTX, CAZ	6 (4)	1	69.8% (30/43)	0.75
2	CIP, AMP, GEN, S, TE, AZM	6 (5)	1		0.75
3	CIP, AMP, S, TE, AZM, CAZ	6 (5)	1		0.75
4	CIP, AMP, CTX, AZM, CAZ	5 (4)	4		0.62
5	AMP, TE, CTX, AZM, CAZ	5 (4)	1		0.62
6	AMP, GEN, CTX, AZM, CAZ	5 (4)	1		0.62
7	CIP, AMP, S, CTX, CAZ	5 (4)	1		0.62
8	AMP, S, CTX, AZM, CAZ	5 (4)	1		0.62
9	AMP, CTX, AZM, CAZ	4 (3)	1		0.5
10	CIP, AMP, CTX, CAZ	4 (3)	1		0.5
11	CIP, AMP, AZM, CAZ	4 (4)	5		0.5
12	AMP, TE, AZM, CAZ	4 (4)	1		0.5
13	AMP, GEN, CTX, CAZ	4 (3)	1		0.5
14	CIP, AMP, S, CAZ	4 (4)	2		0.5
15	AMP, GEN, CAZ	3 (3)	1		0.37
16	CIP, AMP, CAZ	3 (3)	6		0.37
17	AMP, AZM, CAZ	3 (3)	1		0.37
18	AMP, CTX, CAZ	3 (2)	4	Non‐MDR	0.37
19	AMP, CAZ	2 (2)	8	Non‐MDR	0.25
20	CIP, AMP	2 (2)	1	Non‐MDR	0.25

Abbreviations: AMP, ampicillin; CAZ, ceftazidime; CIP, ciprofloxacin; CTX, cefotaxime; GEN, gentamicin; MA, multiple antibiotic resistance; MDR, multidrug‐resistant; S, streptomycin; TE, tetracycline.

### 3.3 ESBL‐Producing *Escherichia coli*

In the phenotypic assay, eight isolates (18.6%, CI_95%_: 9.7; 32.6) were confirmed as ESBL‐EC. Shrimp samples had higher (*p* > 0.05) ESBL‐EC (3/11, 27.0%, CI_95%_: 9.8; 56.7) than in sea fish (4/23, 17.4%, CI_95%_: 6.9; 37.1) and crabs (1/9, 11.1%, CI_95%_: 0.6; 43.5). All ESBL‐EC isolates showed 100% resistance to ampicillin, cefotaxime, and ceftazidime, followed by ciprofloxacin, azithromycin (75%, CI_95%_: 40.9; 95.6), and streptomycin, gentamicin, and tetracycline (12.5%, CI_95%_: 0.6; 47.1). All the ESBL‐EC isolates were MDR (Table 4). PCR revealed that ESBL‐EC isolates contain genes conferring resistance to beta‐lactam antibiotics, for example, β‐lactamase genes *bla*_TEM_ (75%, 6/8, CI_95%_: 40.9; 95.6), *bla*_SHV_ (87.5%, 7/8, CI_95%_: 52.9; 99.4), and *bla*_CTX‐M_ (50%, 4/8, CI_95%_: 21.5; 78.5). Moreover, four isolates harbored all three tested beta‐lactamase genes (Table 4).

**Table 4:** Properties of ESBL‐producing *E. coli* isolated from seafood samples.

Isolates no.	Resistance pattern	MDR	β ‐lactamase genes
1	CIP, AMP, AZM, CTX, CAZ	100%	*bla*_TEM_, *bla*_SHV_, *bla*_CTX‐M_
2	CIP, AMP, AZM, CTX, CAZ		*bla*_TEM_, *bla*_SHV_, *bla*_CTX‐M_
3	AMP, TE, AZM, CTX, CAZ		*bla*_SHV_
4	CIP, AMP, CTX, CAZ		*bla*_TEM_
5	CIP, AMP, AZM, CTX, CAZ		*bla*_TEM_, *bla*_SHV_, *bla*_CTX‐M_
6	AMP, S, AZM, CTX, CAZ		*bla*_TEM_, *bla*_SHV_
7	AMP, GEN, AZM, CTX, CAZ		*bla*_TEM_, *bla*_SHV_, *bla*_CTX‐M_
8	CIP, AMP, S, CTX, CAZ		*bla*_SHV_

Abbreviations: AMP, ampicillin; CAZ, ceftazidime, CIP, ciprofloxacin, CTX, cefotaxime; GEN, gentamicin; MDR, multidrug‐resistant; S, streptomycin; TE, tetracycline.

## 4 Discussion

The findings of this study provide crucial insights into the prevalence, AMR patterns, and genetic characteristics of *E. coli* in raw seafood, emphasizing its implications for public health and food safety. Seafood, a vital component of the global diet, is susceptible to microbial contamination, particularly with *E. coli*, which can lead to foodborne diseases and the spread of AMR. This study highlights the magnitude of these issues, focusing on the occurrence of *E. coli*, its resistance patterns, and the prevalence of ESBL‐producing strains.

In the present study, *E. coli* was isolated from different raw seafood samples like shrimp, tuna fish, pomfret fish, crab, and tiger prawn. The prevalence rates varied among different seafood types, with tuna exhibiting the highest occurrence, followed by pomfret fish, crab, and shrimp. The variation in *E. coli* occurrence across species may be attributed to differences in their biological and ecological characteristics (Jang et al. 2017). Tuna, pelagic fish, and pomfret, often sourced from tropical waters, might be exposed to polluted environments, increasing the likelihood of bacterial contamination (Pan et al. 2024). Crustaceans like shrimp and crabs, being bottom dwellers and filter feeders, can accumulate bacteria from sediments and water, further contributing to their contamination levels (Behringer and Duermit‐Moreau 2021).

In this study, 42.2% of the samples tested positive for contamination with *E. coli*, which aligns with findings from a previous study (44.1%) (Odumosu et al. 2021). However, the lower prevalence of *E. coli* in seafood samples was recorded at 18.7% by Dumen et al. (2020) and 17.7% by Celik et al. (2023). Moreover, several previous studies also detected *E. coli* in seafood samples at various prevalence rates (Khan et al. 2024; Faridullah et al. 2016; Prakasan et al. 2022; Vásquez‐García et al. 2019; Barbosa et al. 2016; Singh et al. 2020). The detection of *E. coli* in seafood samples raises concerns about water quality and hygiene practices during harvesting, processing, and handling. Contaminated water, particularly in aquaculture, serves as a primary source of *E. coli*, suggesting a need for stringent monitoring of water quality (Cho et al. 2022).

Moreover, the notably higher *E. coli* prevalence observed in sea fish (63.9%) compared to shrimp and crabs may be influenced by several ecological and postharvest handling factors. Unlike shrimp and crabs, which are often farmed in controlled environments or caught in nearshore waters, sea fish are typically harvested from open marine environments, where exposure to fecal contamination from untreated sewage discharge, river runoff, or marine pollution is more likely. Coastal and estuarine zones—where many sea fish are captured—often receive wastewater effluents, particularly in densely populated coastal regions of Bangladesh, potentially increasing the microbial load. Also, sea fish are stored on ice but not gutted or processed immediately, allowing for potential bacterial migration from the surface to internal tissues.

Antimicrobial resistance is one of the most talked‐about issues in the world today. As far as we know, this study represents the first documented detection of MDR and ESBL‐EC in raw seafood from Bangladesh. The observed AMR patterns are alarming, as all 43 isolates exhibited resistance to ampicillin, while high resistance levels were also found against ceftazidime and ciprofloxacin. Resistance to other antibiotics, such as azithromycin, cefotaxime, and streptomycin, further highlights the reduced efficacy of commonly used drugs. Lower resistance rates were observed for gentamicin and tetracycline, although their effectiveness may still be compromised due to intermediate resistance patterns. Previously, Odumosu et al. (2021) and Singh et al. (2020) reported a high prevalence of *E. coli* isolates in seafood that were resistant to different antimicrobial classes, which is likely to be the present findings. Moreover, the presence of antibiotic‐resistant *E. coli* in seafood was reported in several studies (Kumar et al. 2005; Matyar et al. 2008; Ryu et al. 2012; Watkinson et al. 2007). The occurrence of MDR isolates in 67.44% of samples is a critical issue. The presence of MDR isolates in seafood‐associated *E. coli* poses a potential public health risk. The MAR indices, ranging from 0.25 to 0.75, indicate a high risk of contamination with bacteria exposed to antibiotics in their environment. These findings are consistent with global reports of increasing AMR in foodborne pathogens due to the misuse and overuse of antibiotics in aquaculture and veterinary practices (Odumosu et al. 2021; Singh et al. 2020). The significant correlation between resistance patterns of azithromycin and tetracycline suggests the possibility of co‐selection mechanisms or linked resistance genes. This finding indicates the complexity of resistance development and highlights the need for molecular studies to unravel the genetic basis of antimicrobial resistance in *E. coli*.

In this study, 18.6% of the *E. coli* isolates were ESBL producers, all exhibiting MDR patterns. ESBL enzymes enable resistance to β‐lactam antibiotics, including penicillins and cephalosporins, making infections caused by such strains more challenging to treat (Husna et al. 2023). However, previous studies reported higher prevalence rates of ESBL‐EC in seafood in India, 71.6% by Singh et al. (2020) and 65.8% by Sivaraman et al. (2020). It might be due to differences in sampling sources, antibiotic usage in aquaculture, and detection methods. Regional variations in environmental contamination, regulatory practices, and methodological approaches could also contribute to the discrepancy. The *bla*_SHV_ gene was detected in 87.5% of ESBL‐EC isolates, surpassing the prevalence of *bla*_TEM_ (75%) and *bla*_CTX‐M_ (50%). A previous study by Singh et al. (Singh et al. 2020) reported the same results, where the ESBL gene *bla*_SHV_ (23.4%) showed dominance compared to *bla*_CTX‐M_ (2.6%). This finding contrasts with global epidemiological patterns, where *bla*_CTX‐M_ has emerged as the dominant ESBL gene family in recent years (Yu et al. 2024). The lower detection rate of *bla*_CTX‐M_ in our isolates may reflect unique local plasmid dynamics, such as the regional circulation of specific plasmid types that preferentially harbor *bla*_SHV_ or *bla*_TEM_ genes. Bangladesh's aquaculture and seafood production systems may also exert distinct selective pressures due to widespread but often unregulated antibiotic use, which could favor the persistence of *bla*_SHV_‐carrying plasmids. Additionally, environmental reservoirs, including contaminated surface waters and sediment, may serve as essential sources of SHV‐type ESBLs, especially in areas with high levels of anthropogenic pollution. More comprehensive genomic studies are needed to understand these resistance determinants' mobility, co‐selection, and persistence in seafood‐associated bacterial populations.

While this study provides the first molecular evidence of ESBL‐EC in seafood from Bangladesh, several limitations should be acknowledged. First, the sampling was limited to selected coastal markets in the Chattogram Division, which may not fully represent all seafood production and distribution settings across the country. Thus, geographic and seasonal sampling bias cannot be ruled out. Second, although phenotypic methods and PCR‐based gene detection were used to confirm ESBL production, whole‐genome sequencing (WGS) was not performed. As a result, the full resistome, plasmid content, and genetic contexts of resistance genes could not be explored. Genomic analysis would offer deeper insights into gene transfer mechanisms, clonal relatedness, and potential co‐resistance traits.

## 5 Conclusions

The study emphasizes the significant occurrence of *E. coli* in seafood from coastal markets in Bangladesh, with a high prevalence of MDR and ESBL‐EC isolates. These findings raise important concerns for public health and food safety. The detection of ESBL‐EC in commonly consumed seafood underscores the critical need for coordinated efforts to mitigate AMR in aquatic environments. Therefore, we recommend implementing a routine national surveillance program targeting antimicrobial‐resistant pathogens in seafood, focusing on ESBL producers. Policies should also enforce regulated use of antibiotics in aquaculture, including veterinary oversight and withdrawal periods, to limit the emergence and spread of resistance. Strengthening good aquaculture practices, such as improved water quality management, hygienic handling, and biosecurity protocols, can reduce contamination at the production level. Additionally, consumer education campaigns focused on proper seafood handling, cooking, and awareness of AMR risks are essential. Public health authorities should collaborate with fishery stakeholders to disseminate best practices. Finally, future research should expand sampling geographically and seasonally and incorporate WGS to gain deeper insights into resistance gene transmission and environmental reservoirs.

## Author Contributions

**Zannatul Firdous:** methodology, software, data curation, investigation, formal analysis, visualization, writing – original draft. **Md. Saiful Islam:** methodology, software, data curation, investigation, formal analysis, visualization, writing – original draft, writing – review and editing. **Md. Ashek Ullah:** methodology, investigation. **Md. Liton Rana:** methodology, investigation, visualization, software. **Farhana Binte Ferdous:** software, formal analysis, investigation, writing – original draft, data curation. **Al‐Muksit Mohammad Taufiquer Rahman:** writing – review and editing, supervision. **Jayedul Hassan:** supervision, writing – review and editing. **Md. Tanvir Rahman:** conceptualization, supervision, resources, project administration, writing – review and editing, funding acquisition, validation.

## Ethics Statement

The ethics committee of Bangladesh Agricultural University, Mymensingh, Bangladesh, reviewed and approved all protocols and methodologies associated with this study (AWEEC/BAU/2023(25)).

## Conflicts of Interest

The authors declare no conflicts of interest.

## Data Availability

All relevant data supporting the findings of this study are provided within the manuscript.

## References

[mbo370023-bib-0001] Ahmed, I. , M. B. Rabbi , and S. Sultana . 2019. “Antibiotic Resistance in Bangladesh: A Systematic Review.” International Journal of Infectious Diseases 80: 54–61. 30634043 10.1016/j.ijid.2018.12.017

[mbo370023-bib-0002] Barbosa, L. J. , L. F. Ribeiro , L. F. Lavezzo , M. M. C. Barbosa , G. A. M. Rossi , and L. A. Do Amaral . 2016. “Detection of Pathogenic *Escherichia coli* and Microbiological Quality of Chilled Shrimp Sold in Street Markets.” Letters in Applied Microbiology 62, no. 5: 372–378. 26960181 10.1111/lam.12562

[mbo370023-bib-0003] Bauer, A. W. , W. M. M. Kirby , J. C. Sherris , and M. Turck . 1966. “Antibiotic Susceptibility Testing by a Standardized Single Disk Method.” American Journal of Clinical Pathology 45: 493–496. 5325707

[mbo370023-bib-0004] Beauchamp, C. S. , and J. N. Sofos . 2010. “Diarrheagenic *Escherichia coli* .” In Pathogens and Toxins in Foods, edited by V. K. Juneja and J. N. Sofos , 71–94. ASM Press.

[mbo370023-bib-0005] Behringer, D. C. , and E. Duermit‐Moreau . 2021. “Crustaceans, One Health and the Changing Ocean.” Journal of Invertebrate Pathology 186: 107500. 33144148 10.1016/j.jip.2020.107500

[mbo370023-bib-0006] Brauge, T. , J. Mougin , T. Ells , and G. Midelet . 2024. “Sources and Contamination Routes of Seafood With Human Pathogenic Vibrio Spp.: A Farm‐to‐Fork Approach.” Comprehensive Reviews in Food Science and Food Safety 23, no. 1: e13283. 38284576 10.1111/1541-4337.13283

[mbo370023-bib-0007] Brown, L. D. , T. T. Cai , and A. DasGupta . 2001. “Interval Estimation for a Binomial Proportion.” Statistical Science 16, no. 2: 101–133.

[mbo370023-bib-0008] CDC . 2025. Antibiotics Tested by NARMS. https://www.cdc.gov/narms/about/index.html.

[mbo370023-bib-0009] Celik, B. , B. Ergul , A. Kekec , et al. 2023. “Beta‐Lactam, Aminoglycoside, and Quinolone Resistance in *Escherichia coli* Strains Isolated From Shrimps and Mussels in the Marmara Sea.” Veterinární medicína 68, no. 5: 208–217. 37982027 10.17221/105/2022-VETMED PMC10581531

[mbo370023-bib-0010] Chen, S. , S. Zhao , D. G. White , et al. 2004. “Characterization of Multiple‐Antimicrobial‐Resistant Salmonella Serovars Isolated From Retail Meats.” Applied and Environmental Microbiology 70, no. 1: 1–7. 14711619 10.1128/AEM.70.1.1-7.2004 PMC321239

[mbo370023-bib-0011] Cho, K. H. , J. Wolny , J. A. Kase , T. Unno , and Y. Pachepsky . 2022. “Interactions of *E. coli* With Algae and Aquatic Vegetation in Natural Waters.” Water Research 209: 117952. 34965489 10.1016/j.watres.2021.117952

[mbo370023-bib-0012] CLSI . 2022. M100‐S32; Performance Standards for Antimicrobial Susceptibility Testing. Clinical and Laboratory Standards Institute: Wayne, PA, USA, 2022.

[mbo370023-bib-0013] Costa, R. A. 2013. “ *Escherichia coli* in Seafood: A Brief Overview.” Advances in Bioscience and Biotechnology 4: 450–454.

[mbo370023-bib-0014] Department of Fisheries . 2023. “Year Book of Fisheries Statistics of Bangladesh 2022–23.” http://fisheries.portal.gov.bd/site/download/2ee50811-cc90-4eca-9150-1fb4ad609b21.

[mbo370023-bib-0015] Department of Fisheries . 2024. “Bangladesh International Aquaculture and Seafood Show 2024.” https://fisheries.portal.gov.bd/sites/default/files/files/fisheries.portal.gov.bd/page/be14e001_cdb6_4778_b5e7_f191b900e3d3/2024-06-12-07-41-2757cda5c8796a042b920daafcdd9d6c.pdf.

[mbo370023-bib-0016] Dumen, E. , G. Ekici , S. Ergin , and G. M. Bayrakal . 2020. “Presence of Foodborne Pathogens in Seafood and Risk Ranking for Pathogens.” Foodborne Pathogens and Disease 17, no. 9: 541–546. 32175783 10.1089/fpd.2019.2753

[mbo370023-bib-0017] Faber, T. A. , P. J. Bechtel , D. C. Hernot , et al. 2010. “Protein Digestibility Evaluations of Meat and Fish Substrates Using Laboratory, Avian, and Ileally Cannulated Dog Assays.” Journal of Animal Science 88: 1421–1432. 20023140 10.2527/jas.2009-2140

[mbo370023-bib-0018] Faridullah, M. , V. C. Roy , and U. J. Lithi . 2016. “Prevalence of Salmonella and *Escherichia coli* Contamination in Shrimp (*Penaeus monodon*) Farms, Depots and Processing Plants in Different Areas of Bangladesh.” Asian Journal of Medical and Biological Research 2, no. 2: 171–176.

[mbo370023-bib-0019] Ferdous, F. B. , M. S. Islam , M. A. Ullah , et al. 2023. “Antimicrobial Resistance Profiles, Virulence Determinants, and Biofilm Formation in Enterococci Isolated From Rhesus Macaques (*Macaca mulatta*): A Potential Threat for Wildlife in Bangladesh?” Animals: An Open Access Journal From MDPI 13, no. 14: 2268. 37508046 10.3390/ani13142268 PMC10376288

[mbo370023-bib-0020] Global Seafoods . 2025. “Seafood Market Forecast: Trends to Expect in 2025.” https://globalseafoods.com/blogs/news/seafood-market-forecast-trends-to-expect.

[mbo370023-bib-0021] Haque, Z. F. , M. S. Islam , A. A. M. Sabuj , et al. 2023. “Molecular Detection and Antibiotic Resistance of Vibrio Cholerae, *Vibrio parahaemolyticus*, and Vibrio Alginolyticus From Shrimp (*Penaeus monodon*) and Shrimp Environments in Bangladesh.” Aquaculture Research 2023, no. 1: 1–11.

[mbo370023-bib-0022] Hassen, B. , A. Jouini , M. Elbour , S. Hamrouni , and A. Maaroufi . 2020. “Detection of Extended‐Spectrum β‐Lactamases (ESBL) Producing Enterobacteriaceae From Fish Trapped in the Lagoon Area of Bizerte, Tunisia.” BioMed Research International 2020, no. 1: 7132812. 32596358 10.1155/2020/7132812 PMC7303757

[mbo370023-bib-0023] Hitchins, A. D. , P. Feng , W. D. Watkins , S. R. Rippey , and L. A. Chandler . 1998. *Escherichia coli* and the Coliform Bacteria.” *In Bacteriological Analytical Manual*, 8th ed. AOAC International.

[mbo370023-bib-0024] Husna, A. , M. M. Rahman , A. T. M. Badruzzaman , et al. 2023. “Extended‐Spectrum β‐Lactamases (ESBL): Challenges and Opportunities.” Biomedicines 11, no. 11: 2937. 38001938 10.3390/biomedicines11112937 PMC10669213

[mbo370023-bib-0025] Huss, H. H. 1993. Assurance of Seafood Quality. FAO Fisheries Technical Paper No. 334, FAO, Rome, 169.

[mbo370023-bib-0026] Islam, M. S. , M. A. Sobur , S. Rahman , et al. 2022. “Detection of *bla* _ *TEM* _, *bla* _ *CTX‐M* _, *bla* _ *CMY* _, and *bla* _ *SHV* _ Genes Among Extended‐Spectrum Beta‐Lactamase‐Producing *Escherichia coli* Isolated From Migratory Birds Travelling to Bangladesh.” Microbial Ecology 83: 942–950. 10.1007/s00248-021-01803-x. 34312710 PMC8313370

[mbo370023-bib-0027] Jang, J. , H. G. Hur , M. J. Sadowsky , M. N. Byappanahalli , T. Yan , and S. Ishii . 2017. “Environmental *Escherichia coli*: Ecology and Public Health Implications—A Review.” Journal of Applied Microbiology 123, no. 3: 570–581. 28383815 10.1111/jam.13468

[mbo370023-bib-0028] Khan, M. , M. M. Rahman , S. I. Paul , and J. A. Lively . 2024. “Detection of Pathogenic Bacteria in Retailed Shrimp From Bangladesh.” Food Science & Nutrition 12, no. 9: 6379–6388. 39554329 10.1002/fsn3.4260 PMC11561818

[mbo370023-bib-0029] Krumperman, P. H. 1983. “Multiple Antibiotic Resistance Indexing of *Escherichia coli* to Identify High‐Risk Sources of Fecal Contamination of Foods.” Applied and Environmental Microbiology 46, no. 1: 165–170. 6351743 10.1128/aem.46.1.165-170.1983 PMC239283

[mbo370023-bib-0030] Kumar, H. S. , A. Parvathi , I. Karunasagar , and I. Karunasagar . 2005. “Prevalence and Antibiotic Resistance of *Escherichia coli* in Tropical Sea‐Food.” World Journal of Microbiology and Biotechnology 21: 619–623.

[mbo370023-bib-0031] Kümmerer, K. 2009. “Antibiotics in the Aquatic Environment—A Review—Part I.” Chemosphere 75, no. 4: 417–434. 19185900 10.1016/j.chemosphere.2008.11.086

[mbo370023-bib-0032] Loest, D. , F. C. Uhland , K. M. Young , et al. 2022. “Carbapenem‐Resistant *Escherichia coli* From Shrimp and Salmon Available for Purchase by Consumers in Canada: A Risk Profile Using the Codex Framework.” Epidemiology and Infection 150: 148. 10.1017/S0950268822001030 PMC9386791 35968840

[mbo370023-bib-0033] Magiorakos, A. P. , A. Srinivasan , R. B. Carey , et al. 2012. “Multidrug‐Resistant, Extensively Drug‐Resistant and Pandrug‐Resistant Bacteria: An International Expert Proposal for Interim Standard Definitions for Acquired Resistance.” Clinical Microbiology and Infection 18, no. 3: 268–281. 21793988 10.1111/j.1469-0691.2011.03570.x

[mbo370023-bib-0034] Matyar, F. , A. Kaya , and S. Dincer . 2008. “Antibacterial Agents and Heavy Metal Resistance in Gram‐Negative Bacteria Isolated From Seawater, Shrimp and Sediment in Iskenderun Bay, Turkey.” Science of the Total Environment 407: 279–285. 18804847 10.1016/j.scitotenv.2008.08.014

[mbo370023-bib-0035] Odumosu, B. T. , H. I. Obeten , and T. A. Bamidele . 2021. “Incidence of Multidrug‐Resistant *Escherichia coli* Harbouring bla TEM and TET A Genes Isolated From Seafoods in Lagos Nigeria.” Current Microbiology 78, no. 6: 2414–2419. 33961094 10.1007/s00284-021-02511-y

[mbo370023-bib-0036] Omati, A. , K. Davari , and B. Shokrolahi . 2016. “Genotyping of *E. coli* Isolated From Urinary Tract Infection Patients Containing B‐Lactamase Resistance Gene CTX‐M Group 1 in Sanandaj Medical Health Centers.” American Journal of Molecular Biology 6: 159–169.

[mbo370023-bib-0037] Pan, B. , J. Zhu , Q. Lin , Z. Geng , F. Wu , and Y. Zhang . 2024. “Study on the Catch, Bycatch and Discard of Chinese Pelagic Longline Fisheries in the Atlantic Ocean.” Aquaculture and Fisheries 9, no. 2: 280–286.

[mbo370023-bib-0038] Prakasan, S. , M. Lekshmi , P. Ammini , A. K. Balange , B. B. Nayak , and S. H. Kumar . 2022. “Occurrence, Pathogroup Distribution and Virulence Genotypes of *Escherichia coli* From Fresh Seafood.” Food Control 133: 108669.

[mbo370023-bib-0039] Robobank . 2025. “Global Animal Protein Outlook 2025.” https://www.rabobank.com/knowledge/q011409742-global-animal-protein-outlook-2025.

[mbo370023-bib-0040] Ryu, S. H. , S. G. Park , S. M. Choi , et al. 2012. “Antimicrobial Resistance and Resistance Genes in *Escherichia coli* Strains Isolated From Commercial Fish and Seafood.” International Journal of Food Microbiology 152: 14–18. 22071288 10.1016/j.ijfoodmicro.2011.10.003

[mbo370023-bib-0041] Singh, A. S. , B. B. Nayak , and S. H. Kumar . 2020. “High Prevalence of Multiple Antibiotic‐Resistant, Extended‐Spectrum β‐Lactamase (ESBL)‐Producing *Escherichia coli* in Fresh Seafood Sold in Retail Markets of Mumbai, India.” Veterinary Sciences 7, no. 2: 46. 32316123 10.3390/vetsci7020046 PMC7356741

[mbo370023-bib-0042] Sivaraman, G. K. , S. Sudha , K. H. Muneeb , B. Shome , M. Holmes , and J. Cole . 2020. “Molecular Assessment of Antimicrobial Resistance and Virulence in Multi‐Drug Resistant ESBL‐Producing *Escherichia coli* and *Klebsiella pneumoniae* From Food Fishes, Assam, India.” Microbial Pathogenesis 149: 104581. 33080358 10.1016/j.micpath.2020.104581

[mbo370023-bib-0043] Surendraraj, A. , N. Thampuran , and T. C. Joseph . 2010. “Molecular Screening, Isolation, and Characterization of Enterohemorrhagic *Escherichia coli* O157:H7 From Retail Shrimp.” Journal of Food Protection 73: 97–103. 20051211 10.4315/0362-028x-73.1.97

[mbo370023-bib-0044] Ullah, M. A. , M. S. Islam , F. B. Ferdous , M. L. Rana , J. Hassan , and M. T. Rahman . 2024. “Assessment of Prevalence, Antibiotic Resistance, and Virulence Profiles of Biofilm‐Forming Enterococcus Faecalis Isolated From Raw Seafood in Bangladesh.” Heliyon 10, no. 20: e39294. 39640770 10.1016/j.heliyon.2024.e39294 PMC11620263

[mbo370023-bib-0045] Ullah, M. A. , M. S. Islam , M. L. Rana , et al. 2023. “Resistance Profiles and Virulence Determinants in Biofilm‐Forming Enterococcus Faecium Isolated From Raw Seafood in Bangladesh.” Pathogens 12, no. 9: 1101. 37764909 10.3390/pathogens12091101 PMC10535238

[mbo370023-bib-0046] Vásquez‐García, A. , A. P. S. C. de Oliveira , J. E. Mejia‐Ballesteros , et al. 2019. “ *Escherichia coli* Detection and Identification in Shellfish From Southeastern Brazil.” Aquaculture 504: 158–163.

[mbo370023-bib-0047] Wang, R. F. , W. W. Cao , and C. E. Cerniglia . 1996. “PCR Detection and Quantitation of Predominant Anaerobic Bacteria in Human and Animal Fecal Samples.” Applied and Environmental Microbiology 62: 1242–1247. 8919784 10.1128/aem.62.4.1242-1247.1996 PMC167889

[mbo370023-bib-0048] Watkinson, A. J. , G. B. Micalizzi , G. M. Graham , J. B. Bates , and S. D. Costanzo . 2007. “Antibiotic‐Resistant *Escherichia coli* in Wastewaters, Surface Waters, and Oysters From an Urban Riverine System.” Applied and Environmental Microbiology 73: 5667–5670. 17616617 10.1128/AEM.00763-07 PMC2042091

[mbo370023-bib-0049] WHO . 2014. “Antimicrobial Resistance: Global Report on Surveillance.” https://www.who.int/publications/i/item/WHO-HSE-PED-AIP-2014.2.

[mbo370023-bib-0050] Yang, J. , C. Wang , C. Shu , et al. 2013. “Marine Sediment Bacteria Harbor Antibiotic Resistance Genes Highly Similar to Those Found in Human Pathogens.” Microbial Ecology 65, no. 4: 975–981. 23370726 10.1007/s00248-013-0187-2

[mbo370023-bib-0051] Yu, K. , Z. Huang , Y. Xiao , X. Bai , H. Gao , and D. Wang . 2024. “Epidemiology and Molecular Characterization of CTX‐M‐Type ESBLs Producing *Escherichia coli* Isolated From Clinical Settings.” Journal of Global Antimicrobial Resistance 36: 181–187. 38072240 10.1016/j.jgar.2023.11.013

